# ScriptingRT: A Software Library for Collecting Response Latencies in Online Studies of Cognition

**DOI:** 10.1371/journal.pone.0067769

**Published:** 2013-06-21

**Authors:** Thomas W. Schubert, Carla Murteira, Elizabeth C. Collins, Diniz Lopes

**Affiliations:** Department of Psychology, University of Oslo, Oslo, Norway; 2 Centro de Investigação e Intervenção Social, Instituto Universitário de Lisboa (ISCTE-IUL), Lisboa, Portugal; National Microelectronics Center, Spain

## Abstract

ScriptingRT is a new open source tool to collect response latencies in online studies of human cognition. ScriptingRT studies run as Flash applets in enabled browsers. ScriptingRT provides the building blocks of response latency studies, which are then combined with generic Apache Flex programming. Six studies evaluate the performance of ScriptingRT empirically. Studies 1–3 use specialized hardware to measure variance of response time measurement and stimulus presentation timing. Studies 4–6 implement a Stroop paradigm and run it both online and in the laboratory, comparing ScriptingRT to other response latency software. Altogether, the studies show that Flash programs developed in ScriptingRT show a small lag and an increased variance in response latencies. However, this did not significantly influence measured effects: The Stroop effect was reliably replicated in all studies, and the found effects did not depend on the software used. We conclude that ScriptingRT can be used to test response latency effects online.

## Introduction

There are two pervasive current trends in how to collect data in psychological studies: One is to take advantage of the Internet and collect data using online methods of various kinds, especially online questionnaires. This allows easier access to traditional participant samples, extension of research to hard-to-reach samples, and the adaptation of items to previous answers. The other trend is the long-standing but ever-increasing tendency to use response latencies as indices of cognitive processing. Such measures are utilized to examine both individual differences between participants, and differences elicited by experimental manipulations [1]–[3].

However, with a few notable exceptions, these two trends have not been merged: Only a fraction of online research measures response latencies to individual trials that last around a second or less. The reasons are of a rather technical nature, and repeated attempts to bridge the gulf demonstrate the need, while still falling short in many ways. The few projects that have bridged the gulf, however, show the great potential of online response latency collection.

In the present work, we offer a fresh approach to the problem. We design, implement, and evaluate an approach to measuring response latencies online that is based completely on open source or free technology, namely Adobe Flash files created with Apache Flex [4] (formerly Adobe Flex), using concepts that are inspired by the free response latency software DMDX [5]. The software that we developed is itself open source and modular, inviting contributions from other laboratories and researchers.

## Previous Approaches to Online Response Latency Measurement

The typical response latency study presents the participant with a number of trials, somewhere between 50 and 300, and asks for as rapid responses as possible. The kind of stimuli in a trial and the task for the participant vary widely, and consequently the response latencies can vary immensely, from ca. 400 to 2000****ms for most tasks. The factors of interest are typically manipulated within participant, and latency differences in the literature range from a few to dozens of milliseconds.

In order to participate in a web-based survey, participants typically only need a standard computer with network connection and a recent browser program. In such surveys, the computer is doing little else besides showing the questionnaire and sending back the answers to the server. However, in order to collect response latencies to many individual trials, some kind of program needs to be executed on the participant's computer (i.e., client-side). This is because communication across networks and responses by servers takes time and the amount of time varies, and would introduce too much noise.

Thus, to run a response latency study online, one needs some program that runs on the participant's computer, and that presents the trials and collects the latencies. Several client side technologies have been used to create such programs: JavaScript, Java, Flash, and native (Windows PC or Mac) code that is downloaded. Let us briefly review some such attempts.

### Java Programs

Most attempts to measure response latencies in online studies have been based on Java. A decade ago, Eichstaedt [6] evaluated the performance of latency measurement by Java. Nosek, Banaji, and Greenwald [7] reported large datasets where the Implicit Association Test (IAT), a robust response latency paradigm, was applied online using applets programmed in Java. Eight of the nine reported IATs showed response latency differences between the critical blocks that were between 95 and 301****ms, with *SD*s lower than 224****ms, resulting in Cohen's *d*s between 0.72 and 1.42. Also working with Java, Keller, Gunasekharan, Mayo, and Corley [8] implemented a psycholinguistic study. Using a self-paced reading time paradigm that produces latencies between 1000 and 2000****ms, they replicated a study previously run in the lab. They estimated that their study had the power to detect reaction time differences above 183****ms. Von Bastian, Locher, and Ruflin recently [9] introduced Tatool, a Java-based open-source programming framework for psychological studies, however without an evaluation of its measurement precision.

### Flash Programs

It is worth noting that all recent online IATs run by “Project Implicit” [10] are programmed in Flash, replacing Java. Reimers and Stewart [11] varied whether participants completed a binomial choice paradigm with 30 trials in the lab using a test programmed in C++, in the lab with a Flash program, or with the same Flash program from outside the lab. They found that Flash added a delay of about 30****ms, but no additional standard deviation to the distribution of latencies.

### Scripting Programs: JavaScript and HTML

JavaScript and HTML 5 have been used by Mason [12] to implement a modular open source version of the IAT. No evaluation of that implementation has been published yet. Zwaan and Pecher [13] also relied on standard JavaScript to measure response latencies, but analysed median instead of mean values. Recently, Crump, McDonnell, and Gureckis [14] replicated several reaction time paradigms with JavaScript programs and participants recruited through Amazon MTurk [15]. They found solid replication of the chosen reaction time-based paradigms (Stroop, task-switching, flanker task, Simon, and Posner cuing). They also observed that control over stimulus presentation times could be achieved down to about 80****ms, but not shorter.

### Native Windows Programs

Two originally PC-based software packages designed to collect response times have made efforts to extend their reach to online data collection. The commercial software Inquisit enables running studies online. To participate in such a study, one must download either an executable file directly or one wrapped in Java Web Launch. Similarly, the free software package DMDX [5] offers a remote testing mode where participants must download an executable file. In both cases, the advantage is that well performing code is executed on the client's machine – in fact the same code as is used for desktop testing, except that the machines running this code will vary much more than a well-managed lab, adding error variance [16], [17]. The disadvantage is, in both cases, that participants must trust the source enough to allow the download of executable code. Security concerns, anti-virus software, and browser restrictions make this difficult, and possibly limit these packages to applications where participants know the entity conducting the study well enough (e.g., to students of a university).

### Comparison and Summary

In sum, Java, JavaScript, and Flash all have been used to measure response latencies online. The published evidence suggests that all three can be successfully used, but also that all three can be expected to increase noise in comparison to native programming on a PC, which can serve as an alternative.

The best comparison of these techniques to native programs that we are aware of has been published by Neath et al. [18]. They built a device that allowed the standardized evaluation of a system's latency and programmed a simple task where the screen turned from black to white. The onset of the white screen was detected by a light sensor placed on the screen, which was connected to a solenoid that was placed above the keyboard. When light was detected, the solenoid fired and pressed a button, given the answer in the trial. Using this system, Neath et al. evaluated four different hardware setups of Macintosh computers, and the same procedure using different software. When Neath et al. [18] programmed their task in Java, the average latency of their device was measured as 99.72****ms, with an average *SD* of 6.66****ms. Flash measured an average latency of 91.56****ms, with an average *SD* of 8.14. Javascript measured the reaction time as being on average 88.07****ms, with an average *SD* of 5.94****ms. As a comparison, when measuring with Matlab and Psychtoolbox and synchronized displays (i.e., native software), an average latency of 49.88, average *SD* = 2.63, was obtained. The additional measurement error in Java, Flash and JavaScript seems acceptable for many paradigms.

### ScriptingRT

We developed a software library that supports programming response latency studies in Flash, called ScriptingRT (online at http://reactiontimes.wordpress.com/scriptingrt/). ScriptingRT studies run in a Flash plugin or in a Flash-supporting browser. They are programmed in Apache Flex and then compiled into Flash applications that can be distributed online and embedded in HTML pages.

Flex is a combination of an XML-based markup language (MXML) and a scripting language (ActionScript). Flex started as an Adobe product, but became open source in 2012, hosted by the Apache Foundation. Because ScriptingRT is also released under an open source license, developing ScriptingRT studies is thus completely based on open source and free software. In order to develop ScriptingRT studies, researchers need to install the Flex SDK (available for OS X and Windows), the ScriptingRT library [19], a text editor, a Flash-enabled browser, and a server running PHP on which data are stored.

The philosophy of ScriptingRT is to allow the programming of a response latency study using a simple set of markup tags in an XML file, while at the same time allowing the programming of additional functionality. The ScriptingRT markup tags and their functionality are defined in the ScriptingRT library, which is used to compile the studies into Flash files.

The concepts for this markup language are inspired by, but not identical to, the free response latency software DMDX. Studies are programmed as a combination of blocks, which consist of items; items, for their part, are made up of frames. A frame represents what is displayed on a single screen at one time. An item can consist of one or more frames. For instance, one item can include a blank intertrial interval frame, a frame with an asterisk to signal the next trial, a frame with a prime, and a frame with a target. Frames never change order within an item. Similarly, a block can be made up of one or multiple items. However, the order of items within a block can change (randomization). In their attributes, items save what the correct response should be.

Each of these structures is constructed with XML tags: A frame is created with the markup <Frame></Frame>, items are constructed with <Item></Item>, and blocks are constructed with <TestPart></TestPart>. Blocks that do not collect response latencies (e.g., for instructions) are constructed with <Part></Part>.

These XML tags are defined by the ScriptingRT library. In combination with such ScriptingRT-specific tags, standard Flex tags can be used. For instance, <Text> </Text> would be used to present text within a frame. As a result, every ScriptingRT code is a mix of tags from the ScriptingRT library and standard Flex tags. An item from a Stroop task could be coded as:

<TestPart id = "stroop" positive = "keyboard.Q.press" negative = "keyboard.P.press" scramble = "1" backgroundColor = "0xffffff" color = "0x000000">

<Item id = "i1" type = "+">

<Frame response = "Time.2000" protocol = "false">

<mx:Text verticalCenter = "0" horizontalCenter = "0" fontSize = "48" text = "+"/>

</Frame>

<Frame id = "if1" protocol = "true">

<mx:Text id = "text100" verticalCenter = "0" horizontalCenter = "0" fontSize = "36" color = "0xff0000" text = "red"/>

</Frame>

</Item>

</TestPart>

**Figure 1:**
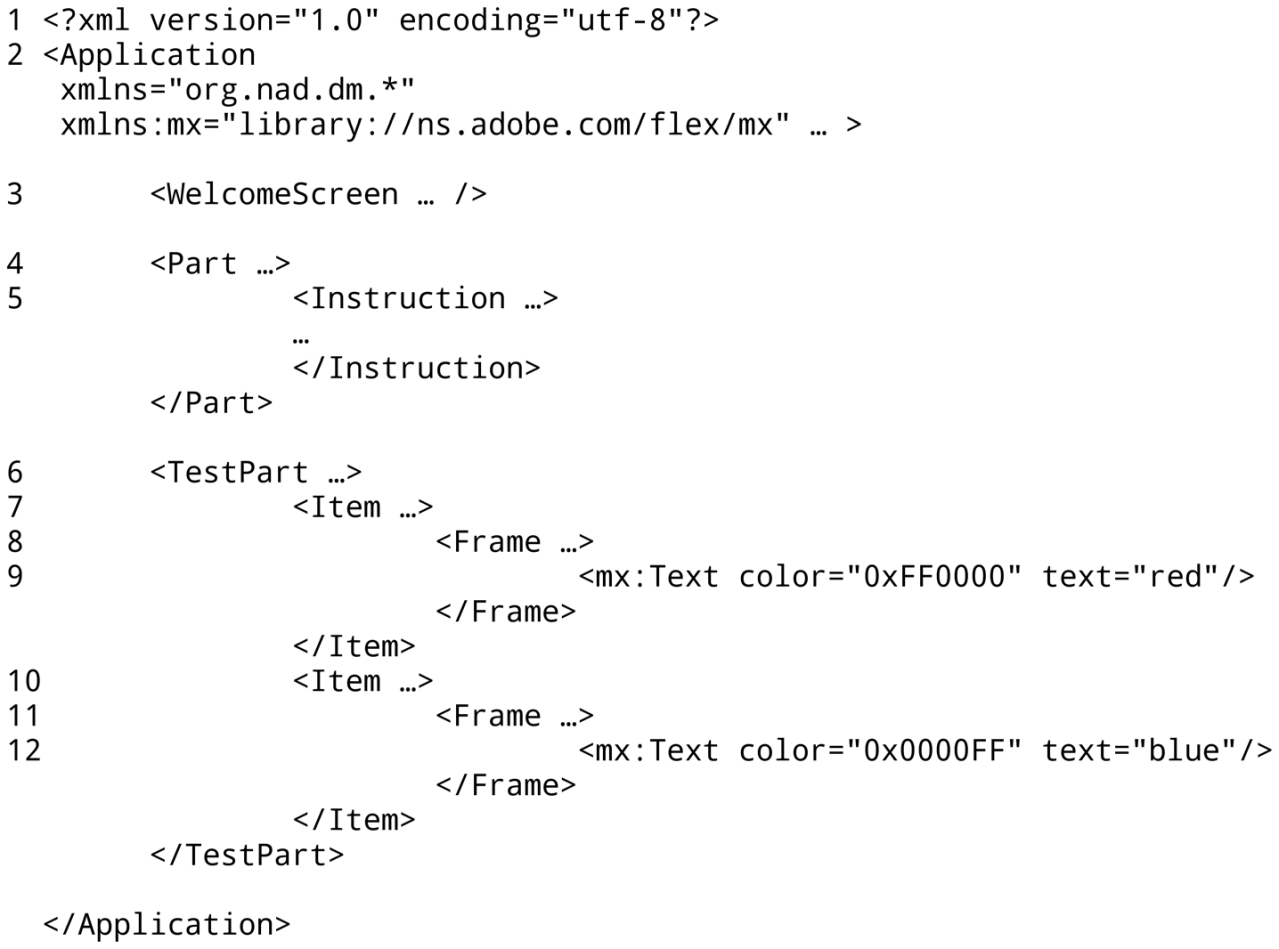
Outline of a Stroop study implemented in ScriptingRT. Excerpt from a ScriptingRT source code file implementing a Stroop paradigm. The tags shown set preferences (tags 1 and 2), create an introductory screen and an instruction screen (3–5) as well as one experimental block with two items (6–12). Tags 1 and 2 are general Apache Flex components. Tags 9 and 12 also embed a general Flex object, a <Text/> object (referenced in the Application tag with the prefix mx). All other tags are part of the ScriptingRT library (referenced in the Application tag).

In this example, the <TestPart> tag sets the defaults for this block, including the expected answers (Q and P), randomization of items (scrambling), and default colors for background and text. Within this block one item with two frames is created. The type of the item is marked as positive with a plus sign, which means that the correct answer will be given with the key identified as correct for positive answers (O). The first frame stays on screen for 2000****ms and displays an asterisk, which is realized using a Text element from the regular Flex library. The second frame stays on screen until an answer is given. In this example, all XML tags except the <Text/> tag are defined by ScriptingRT. (That is also why the <Text/> tag has the prefix “mx:”, which points to a previous definition of additional libraries in the opening <Application/> tag, not shown here.) Figure 1 shows an example of the structure of a complete experiment.

Other building blocks of ScriptingRT studies are special tags for instructions, feedback, and branching. A full list is given in Table 1. Except branching, all of these elements can be used without coding additional ActionScript. More information on the ScriptingRT library is available in the manual [19]. Additional capabilities that are neither in the standard nor the ScriptingRT library can be added by either importing other Flex libraries, or by writing ActionScript programs that are embedded in the XML file. ScriptingRT can contain any Flex object, such as text, html-coded text, vector graphics, images, or sound.

**Table 1:** Overview of main tags and attributes introduced by the ScriptingRT library.

Tag	Explanation
<WelcomeScreen/>	Defines the contents of the first screen, which is equipped with a button to be pressed
<Part/>	Creates a block of items and instructions that are not collecting latencies
<Instruction/>	Displays an instruction
<TestPart/>	Creates a block of items that collect responses and latencies
<Item/>	Creates an item that can contain one or more frames
<Frame/>	Creates a frame, the basic unit of stimuli presentation
response	Attribute of <Part/> and <TestPart>, defines the way a frame can end. Can be keyboard event, mouse event, or time event.
positive, negative, noresponse	Attributes of <TestPart/>, define expected positive and negative response, and maximum time for answer
scramble	Defines way of item randomization in a <TestPart/>
outputURL, protocolFormatHead, protocolFormatItem,	Used in the opening <Application/> tag, define location and formatting of results output
debug	Used in the opening <Application/> tag, displays protocol for debugging at run time
finishedButtonValue	Used in the opening <Application/> tag, sets exit message
<repeat/>	Attribute of <TestPart/>, creates frames that are repeated before frames in every item
<Feedback/>, <correct/>, <incorrect/>, <miss/>	Creates feedback frames to be displayed after each item
<branches/>, <Branch/>	Attributes of TestParts, implementing branching between them

For each answered item, ScriptingRT saves the response time and whether the response was correct or incorrect. Latency measurement is implemented with the standard Flex component flash.utils.Timer, and more specifically the flash.utils.getTimer, which measures in milliseconds.

ScriptingRT flash files can exchange data with the HTML page in which they are embedded. After the study is finished, the data are transmitted to a server that receives them using a PHP script. The format is fully customizable; additional information can be appended if programmed (e.g., order of randomized blocks, data transferred from the HTML page).

ScriptingRT focuses on measuring response latencies to specific stimulus presentations. Other response formats, such as text or scales, are available as standard components of Flex and could be incorporated in ScriptingRT studies. However, instead of adding other response formats, we rather envision ScriptingRT as one element of a larger web-based study, where open ended questions and rating scales that do not require latency measurement are implemented in HTML, and only the trial presentation is done with ScriptingRT in Flash. This approach can benefit from advanced HTML survey software.

One factor within Flash that we do not address empirically here is the frame rate specified in the Flex file. We used both the default (24 frames per second) and a modified rate of 60. Our current recommendation is to use 60****Hz.

In sum, a ScriptingRT study is programmed in Flex, compiled into a Flash file, and then embedded in an HTML page where it collects data that are sent back to a server. ScriptingRT studies are a combination of standard Flex and ScriptingRT-specific functionality for response time studies, and can also contain additional Flex libraries for special content, as well as additional ActionScript for special functionality. In combination, these building blocks provide a programming environment for creating many types of response latency studies, plus flexibility for more experienced programmers. The library is ready to be used, and available in source code. It should be noted that the functionality is under development and we hope will be continuously extended. A complete description of the philosophy, syntax, and capabilities of ScriptingRT is available online [19]. In the remainder of this paper, we present empirical investigations where we tested ScriptingRT using online samples, and also in the lab with independent timing equipment.

## Overview of the Current Research

Six studies were run to evaluate the ScriptingRT software and to compare it to standard software packages. Studies 1 through 3 used hardware to evaluate the precision and accuracy of response latencies and presentation times in ScriptingRT. Studies 4 through 6 applied the Stroop paradigm, testing the replicability of the Stroop effect in ScriptingRT and comparing it to other software, both in the lab (Study 5) and online (Studies 4, 6).

We used a variety of hardware and software across the studies, and compared the performance of different software versions where possible. Table 2 gives an overview of the studies.

**Table 2:** Overview of the Studies.

Study	Data Collection	Hardware and Software
1	Timing study with external microcontroller	Arduino Leonardo board connected to Sony Vaio Core i5 laptop, ScriptingRT running in various browsers, and DMDX
2	Timing study with external microcontroller and solenoid	Arduino Uno board interacting with Intel Core i7 desktop computer, ScriptingRT and various other packages
3	Timing study with external microcontroller measuring presentation times	Arduino Uno board measuring Sony Vaio Core i5 laptop, ScriptingRT in Adobe Flash player and Flash plugin in Firefox, and DMDX
4	Online data collection with human participants	Various hardware and flash/browser software programs used by participants
5	Laboratory data collection with human participants	Sony Vaio Core i5 laptop, ScriptingRT running in Firefox with Adobe Flash plugin
6	Online data collection with human participants	Various hardware used by participants

### Ethics Statement

Only Studies 4–6 dealt with human participants. The studies were conducted in concordance with the Ethics Guidelines issued in 2012 by the Scientific Commission (Comissão Científica) of the hosting institution Centro de Investigação e Intervenção Social, Lisboa, Portugal (CIS-IUL). These Ethics Guidelines provide a checklist to decide whether a formal review process is necessary. This checklist indicated that the current studies were exempt from formal ethics review because data were 1) collected anonymously with no pressure to complete, 2) did not involve questions about undesirable personal characteristics, 3) did not involve participants from a population of concern, 4) did not involve deception, 5) did not involve ingesting anything, 6) did not involve invasive measures, 7) did not collect personally identifying information (defined as name, IDs, physical or email addresses, or images), and 8) did not collect potentially endangering information.

All experiments were noninvasive, no false information was provided, and the results were analyzed anonymously. Data were collected sampling only adults. The participants in Study 4 were recruited online through a social network, those from Studies 5 and 6 were recruited at a Portuguese university (in person for Study 5 or through email for Study 6). Thus, all data were collected inside Portugal, the country of the hosting institution. In all three studies, participants read the description and purpose of the study on the initial screen, and were there informed that by proceeding, they consented to participating, but that they could withdraw at any stage of the study. In Study 5, which took place in the laboratory, this was repeated verbally.

## Study 1: Comparing ScriptingRT to Other Software Using Automated Responses with an Emulated Keyboard

We start by examining how much variability in response times is introduced by the software ScriptingRT and Flash. Thus, in Study 1, we removed variance due to human variability and kept differences between trials to a minimum by automatizing the responses to a single stimulus. For this purpose, we employed an Arduino Leonardo Microcontroller board that detected the onset of stimuli on the screen with a light dependent resistor and sent virtual “key strokes” to the computer, emulating a participant's response on a keyboard. The latency of the board's “reactions” was then measured.

### Method

We connected an Arduino Leonardo microcontroller board to a computer by USB. The board can emulate a computer keyboard and send key strokes to the computer that are recognized as coming from a regular keyboard. The Arduino itself was equipped with a TinkerKit light dependent resistor (LDR) sensor, which was placed on the screen of a Vaio Core i5 laptop. The Arduino was programmed such that it checked the state of the LDR continuously. When the reading surpassed the threshold (i.e. was brighter than a certain programmed criteria), the Arduino communicated by USB with the computer, sending a SPACE key stroke, which appeared as a regular press of the space bar on the computer.

We programmed a simple task, which presented 100 trials, in both ScriptingRT and DMDX. The number of trials in this and the following two studies was derived from the typical cognitive science study, which has somewhere between 50 and 300 trials. In each trial, an inter-trial interval with a black screen was followed by the stimulus, a plain white screen, which remained until the press of the space bar was registered. The onset of this white screen was detected by the LDR. The length of time from the display of the white screen to the registration of the key press was measured as the latency. We ran the ScriptingRT task three times on the same laptop: in Chrome 24, Firefox 16, and IE9, all under Windows 7. All browsers relied on the Adobe Flash plugin 11.5.

### Results

The DMDX software set the benchmark in this study. It registered response rates from the Arduino Leonardo between 6.86 and 8.21****ms after the onset of the stimulus, *M* = 7.60, *SD* = .30. Running ScriptingRT, Chrome detected responses between 50 and 97****ms later, *M* = 72.21, *SD* = 6.84. In IE9, response times ranged from 52 to 70****ms, *M* = 60.92, *SD* = 4.93. Firefox registered response times between 50 and 90****ms, *M* = 64.31, *SD* = 6.56.

When running *t*-tests to compare the means, they differed in all cases, *t*s>4.13, *p*s<.001. We compared the standard deviations with Levene's tests. They differed significantly between Firefox and IE9, *F*(1,198) = 14.54, *p*<.001, and obviously between DMDX and the other three measures, but not between Chrome and the other two browsers (Chrome vs. IE9: *F*(198) = 2.72, *p* = .101).

### Discussion

In Study 1 we used a well performing benchmark: DMDX in combination with an Arduino Leonardo board that detected stimulus onset with a light sensor and emulated a keyboard press as a response. DMDX detected this emulated response with a very low standard deviation of less than half a millisecond.

Not surprisingly, ScriptingRT was less precise. In three browsers, measured response latencies had ranges between 18 and 47****ms, and their averages differed significantly. On the other hand, the *SD*s of these responses stayed below 7****ms in all three browsers. That value is comparable to many regular keyboards and standard reaction time software. In addition, the constant added by measuring in ScriptingRT was about 60****ms. This result suggests that researchers using ScriptingRT should thus focus primarily on differences between RTs and be cautious when interpreting absolute latencies.

For researchers using ScriptingRT, knowing the size and the distribution of the offset is useful. In addition, it is instructive to know how this offset is produced. Several separate delays may feed into it. First, ScriptingRT starts measuring the latency as soon as the command to display the stimulus is issued, but the actual presentation on the screen might be delayed. A second possible delay occurs between the registration of the key press by the operating system and the activation of a key press event in the Flash software. A third delay may occur between the firing of that event and the recording of a time stamp in ScriptingRT.

We conclude that not surprisingly, ScriptingRT is less precise in the measurement of latencies than a natively run specialized response latency software program. Furthermore, different browsers result in somewhat different average latencies. Nevertheless, the offset produced by ScriptingRT seems acceptable.

## Study 2: Comparing ScriptingRT to Other Software Using Automated Responses with a Keyboard

Study 1 used an emulated keyboard, and thus could not compare performance with a regular keyboard. That was the goal of Study 2. For this study, we built a machine that detected the appearance of a stimulus (a white screen following a black screen) with a photodiode, and then pressed a response button on an actual keyboard with a solenoid. It thus simulated a human participant in a reaction time task with the aim of getting constant external responses. With this setup, we compared various software packages to ScriptingRT using a standard keyboard [18], [20].

The same machine and procedure was used in previous work on response boxes by our laboratory [21]. In those tests, it was found that both a PST serial response box (connected to the serial port) and a new response device based on an Arduino microcontroller board resulted in standard deviations between 1 and 1.4****ms. This confirms that the machine used here had a rather low variance in its answers. The average reaction time as measured by E-Prime [22] was about 50****ms. This response time was a combination of the time the robot needed to register a change and to fire the solenoid, the pure travel time of the solenoid to the key, plus the time Windows and the software required to register the button press.

### Method

A simple test experiment was programmed in ScriptingRT, E-Prime 2.0.10.178, Inquisit 3.0 [23], DMDX 4 [5], and SuperLab 4.0 [24]. Each trial consisted of a black screen presented for 2000****ms, followed by a white screen, representing the stimulus to which the robot should respond. The white screen remained until a response was detected. As response devices, we used a Microsoft keyboard (model 1047 KU-0459) or a PST Serial Response Box Model 200 (the latter only with E-Prime). Each experiment consisted of 100 trials, and we repeated each two times, resulting in 200 trials. For the sake of simplicity, in this paper we analyzed all 200 trials combined.

All tests were run on the same desktop computer (Intel Core i7, 3.4GHz) with an Nvidia GeForce GTX 560 graphics processor, running at 60****Hz frame rate, using Windows 7 (64-bit), on an Asus VE278 flat screen. The display resolution was set to 1920×1080 pixels, with a refresh rate of 60****Hz and 32 bit of true colors, maximum brightness.

The machine was built using a photodiode to sense stimulus onset, a solenoid to press the button, and an Arduino microcontroller board to connect the two. The photodiode was an Osram BPW 34, the solenoid an Intertec ITS-lz-2560 d-12vdc, and the controller an Arduino Uno, based on an Atmel ATmega328, running at 16****MHz. The Arduino was programmed to consecutively read the input from the photodiode. When a change of brightness was detected on two consecutive readings, the solenoid was fired, pressing a keyboard button positioned below it, and then turned off again after the next change. During the experiment, the machine was only connected to a power source; there was no communication between the computer running the study and the machine [34].

### Results

Table 3 summarizes the response latencies obtained using the machine, for each software and periphery. The benchmark here is the performance using the E-Prime PST serial response box (PreRelease  = 0; we also ran the same test with PreRelease  = 2000, which lead to worse performance. In E-Prime, PreRelease instructs the computer to prepare the next display while still executing the current display. In theory, setting PreRelease to the duration used to present the current display should allow the fastest performance, and this is thus the default in the most recent version of E-Prime. We do not know the reasons for this unexpected result in E-Prime. These data are identical those in Schubert et al. [21])

**Table 3:** Means and SDs of measured response times (in ms) by software (Study 2).

	Descriptives		Comparison of Variances	
Software	*M*	*SD*	*F*(1,198)	*p*
ScriptingRT	92.80	4.21	–	–
E-Prime SRB (PR = 0)	56.91	1.37	131.89	<.001
E-prime SRB (PR = 2000)	56.47	1.85	102.56	<.001
E-prime (PR = 0)	84.58	6.25	12.84	<.001
E-prime (PR = 2000)	70.96	3.30	7.57	.006
DMDX	68.24	3.18	10.75	.001
Inquisit	70.05	3.20	9.78	.002
Superlab	98.18	4.17	<1	.822
InquisitWeb	66.21	2.74	24.04	<.001

*Note.* Last two columns show comparisons of each variance to the variance measured by ScriptingRT (first row). All measures used a keyboard except those labelled SRB, indicating Serial Response Box. PR  =  PreRelease in E-Prime.

E-Prime in combination with a keyboard (and PreRelease  = 2000), Inquisit, DMDX and also the Web version of Inquisit all register somewhat longer overall averages than does E-Prime with a response box, but with acceptable *SD*s between 2.7 and 4****ms. Surprisingly, both E-Prime when using a keyboard and no PreRelease and Superlab produced higher averages and *SD*s larger than 4. ScriptingRT itself registered the machine's response latencies with an average of 93****ms and a *SD* of 4.21 – about 36****ms slower than E-Prime using the response box (the shortest reaction time), and with about three times the *SD*.

We analyzed these reaction times by submitting them to a mixed model with software as a fixed factor. (Note that the mixed model is in this case mathematically identical to a General Linear Model.) This model showed a highly significant effect of software on the average latency, *F*(2786) = 2374, *p*<.001. We used the estimated marginal means to compare the average latency measured by ScriptingRT to each of the other software programs (using SIDAK corrections). The average of ScriptingRT's measurements was significantly different from every other software, all *p*s<.001. Thus, it was significantly slower than all, except Superlab, which it was significantly faster than. We also computed whether the standard deviations of ScriptingRT's measures differed from the *SD*s produced by the other software packages, by computing Levene's tests comparing the respective variances. The last two columns of Table 3 show that ScriptingRT's *SD* is significantly larger than those of every other program except Superlab and E-Prime in one configuration.

### Discussion

Study 2 evaluated ScriptingRT's performance when measuring reaction times in comparison to other software packages. For this purpose, we created a machine that pressed a button in response to a stimulus onset. Previous tests confirmed that measurements with this machine produce standard deviations below 1.5****ms with precise hard- and software [21].

ScriptingRT resulted in both longer response latencies and a larger standard deviation than all other packages except SuperLab and E-Prime in one configuration. Nevertheless, in absolute terms, the *SD* of 4.21 is comparable to what was standard for keyboards for a long time [16]. It is thus clear that any test with ScriptingRT should be well powered and used to assess primarily paradigms with a large effect size. At the same time, the differences between the other tests show that differences are also present between different native software packages (e.g, Superlab vs. DMDX), or can be due to specifics of programming (e.g., the pre-release in E-Prime) and hardware (keyboard vs. response box). Thus, researchers should always be aware that their choice of software, programming, and hardware results in a specific amount of error variance that often can only be evaluated through empirical testing.

## Study 3: Measuring Refresh Rate in ScriptingRT

In addition to the measurement performance of ScriptingRT, it is useful to know how precise the timing of presentations can be. For this purpose, we programmed varied duration, quickly changing presentations in both ScriptingRT and DMDX, and measured the duration of each presentation with an external photodiode. As we anticipate that ScriptingRT will not be used for millisecond-accurate or subliminal presentations, but rather with presentations that have a minimum of about 100****ms, this was our lowest presentation time.

### Method

We programmed ScriptingRT and DMDX (as a comparison) scripts that switched between a white and a black screen 250 times, with varying presentation durations. In ScriptingRT screen presentation durations were 100, 200, or 300****ms. We ran these with the standalone Flash player and also with the plugin running in Firefox. The timing method used in DMDX is based on units of so-called tics – one refresh cycle of the screen. We used a screen with 60****Hz refresh rate, which resulted in single tics that were 16.664****ms long. Each screen was displayed for 1, 2, 3, 4, 5, or 10 tics. All tests were done on a Vaio Core i5 Laptop. The ScriptingRT tasks ran with a display resolution of 1366×768 pixels.

To measure the duration of each black and white screen, we used the photodiode component of the machine used in Study 2. The Arduino microcontroller to which the photodiode was connected registered a change in brightness when two consecutive readings indicated it, and saved the duration in ms between the changes in its internal memory. It did this for 250 changes, and then sent all measurements via the USB connection to the attached PC. In other words, the Arduino measured and recorded the measurements stand-alone and offline during the test, and only transmitted them afterwards.

### Results

Table 4 summarizes the results. The first six lines confirm that the apparatus measured presentation duration rather precisely and with low standard deviations: We obtained absolute differences between 0.03 and 0.21****ms between how long DMDX and the screen ideally should have presented for and what was measured, and *SD*s varied between 1.11 and 2.81****ms.

**Table 4:** Presentation times by DMDX and ScriptingRT (in ms, Study 3).

Software	Target Time	*M*	*SD*
DMDX	16.66 (1 tic)	16.63	1.11
	33.33 (2 tics)	33.20	2.56
	49.99 (3 tics)	49.78	2.17
	66.56 (4 tics)	66.42	2.20
	83.32 (5 tics)	83.20	1.53
	166.64 (10 tics)	166.43	2.81
Flash Standalone Player, 60 Hz refresh rate	100	124.57	10.86
	200	223.43	11.70
	300	323.64	10.45
Flash plugin in Firefox, 24 Hz refresh rate	100	124.84	11.45

*Note.* Measured with an Arduino connected to a photodiode, for 250 switches between a black and a white screen (Study 3).

ScriptingRT produced a rather constant lengthening of about 24****ms in its presentation duration. This was independent of the programmed presentation duration. Likewise, there was a standard deviation of around 11****ms that did not depend on the programmed duration. The plugin and the standalone player did not differ.

### Discussion

In contrast to specialized experimentation software (DMDX), Flash/ScriptingRT adds a constant duration of about 24****ms to each presentation, and the standard deviation of the measured presentation duration was about 11****ms, or 5 times higher. Note that this test switched the screen consecutively between white and black about 250 times, which is probably a rather straining test. Note also that these results might be specific to the display monitor used in this study. Nevertheless, the conclusion is that stimulus presentations in Flash/ScriptingRT cannot be relied upon to be more precise than the above numbers, and that the choice of paradigm should follow these constraints.

## Studies 4–6 Overview: Stroop Task

Studies 1–3 outlined the basic capabilities of ScriptingRT, leading to the conclusion that it will be safely replicating robust paradigms that do not require very short presentation times. The following three studies apply such a paradigm to evaluate and compare ScriptingRT to various other software packages.

We adapted a standard color Stroop task for our evaluation purposes [25]. This is one of the most robust findings in cognitive psychology [26]. We used the version employed by Jostmann and Koole [23].

### Method

We created a Portuguese version of the Stroop task. The stimuli were either words (*vermelho*  =  red, and *azul*  =  blue) or a neutral letter string (XXXX), that appeared either in red or blue script on a white background. Each color word was presented in both colors ten times, and the neutral string was presented in each color ten times, resulting in a total of 60 trials. Trial order was randomized. In addition, there were 10 practice trials. Congruent trials were those where the word named the color it was written in. Incongruent trials were those where the word named the other color.

Each trial consisted of a blank frame (2000****ms), followed by a frame with a central fixation cross “+” (1000****ms), followed by the target, which stayed on screen until a response was detected. Participants were instructed to press Q if the word was written in red and P if was printed in blue. In order to reduce additional variance, the response keys were not counterbalanced.

### Analytical strategy

Jostmann & Koole [23] focused on the Stroop interference alone (i.e., the difference between incongruent and neutral trials) for theoretical reasons. We decided to focus our analyses on the combined interference and facilitation effect (the comparison of congruent and incongruent trials) because a) we are not interested in the difference between interference and facilitation here, b) in preliminary analyses, facilitation effects were much smaller and mostly insignificant, while interference effects were strong and always significant [27]. When comparing congruent and incongruent trials, it is possible to include judged color as another factor (which in our case is confounded with which hand is used to answer). Preliminary analyses showed that this factor did not explain a significant amount of variance here, and we thus also dropped it from our reports for ease of presentation. (Analyses including neutral trials and color as an additional factors can be requested from the first author.)

We report the significance of each statistical test, the 95% confidence interval for the difference, and an effect size. Both the significance tests and confidence intervals were computed by submitting the data to a mixed model (also known as hierarchical linear model) in SPSS 20. In mixed models, individual response latencies are the units of analyses, instead of averaging them to create composite scores, which is the traditional practice. Mixed models have recently been recommended over the standard practice because of more precise and often more conservative testing, and because of enhanced modeling options [28], [29]. However, effect size estimation remains difficult in mixed models. For this reason, after reporting test and confidence intervals estimated from the mixed model, we then averaged response latencies for each participant and computed effect sizes in the traditional manner to report here.

In order to have an *a priori* estimate of the effect size of the Stroop effect, we computed a weighted average of the Stroop interference Jostmann and Koole [23] reported in their Study 1: a difference of 85****ms with an *SD* of 84****ms, a large effect. To replicate such an effect at a significance level α<.05 and with a test power of .80, one would need ten participants. The combined effect of facilitation and interference, which we are going to test, is likely larger. We sampled more than ten participants in each study to assure sufficient power.

## Study 4: Replicating Stroop with ScriptingRT online

### Method

#### Overview and design

Study 4 was conducted online. Participants performed the Stroop task programmed in ScriptingRT with the following design: 3 (target word: blue vs. red vs. xxxx, within) ×2 (color: red vs. blue, within) design.

#### Participants

All participants volunteered to take part in the experiment. Recruiting was done on Facebook from a Portuguese community. After removing cases with missing values, 19 participants remained in the sample (Age: *M* = 28.3, *SD* = 6.3).

#### Materials and procedure

The Flash applet running the ScriptingRT task was 600 pixels high and 800 pixels wide. The frame rate was set to the Flex/Flash default of 24 Hz. The flash applet was embedded in an HTML page. Initial survey instructions requested that participants switch the browser into full screen mode and explained the task: particularly to answer as fast as possible, but also as correctly as possible.

### Results

#### Response latencies

All participants answered more than 48 of the 60 trials correctly. Incorrect responses and latencies above 1600 ms or below 300 ms (together 5% of all trials) were removed from the analyses.

We submitted the response latencies to a mixed model, entering congruency as a fixed effect, and participants as the grouping variable. Congruent trials were answered faster (*M* = 577.32, *SD* = 195.58) than incongruent trials (*M* = 632.59, *SD* = 246.26), showing the classical Stroop effect. This difference was statistically significant, *F*(1,702) = 18.81, *p*<.001.

The traditional way to analyse these data would be to average trials of one type for each participant, and then to subject them to a GLM or equivalent analysis. For comparison purposes, we did this for this sample, averaging for each participant congruent and incongruent trials into two separate scores. When we tested this as a repeated factor in a GLM, we found that congruency had a significant effect, *F*(1,18) = 9.18, *p* = .007.

**Figure 2:**
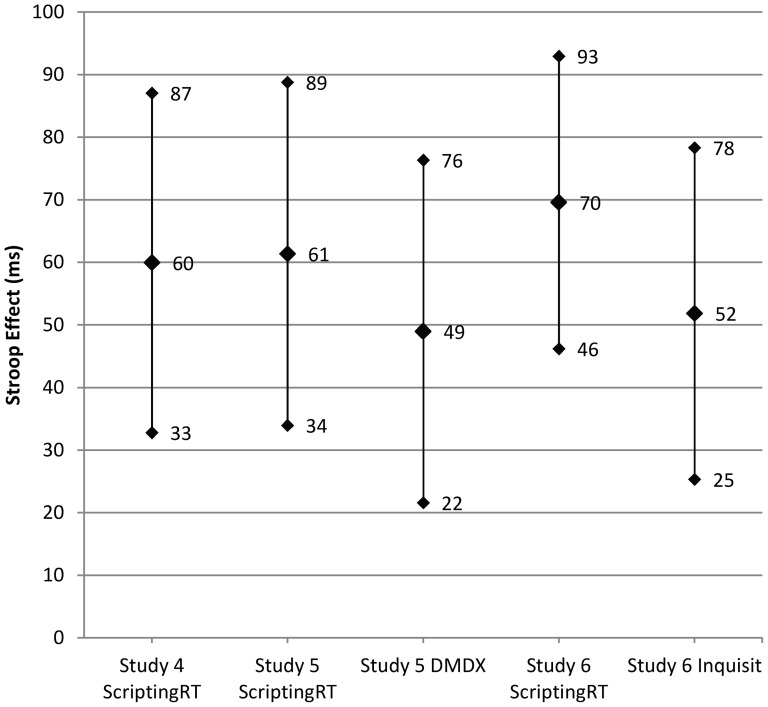
Stroop effects and confidence intervals. Estimated mean Stroop effect (average difference between response latencies in incongruent and congruent trials in ms) and their 95% confidence intervals, obtained in three studies with five samples. Studies 4 and 6 were run online, Study 5 in the laboratory. Software varied within participants in Studies 5, and between participants in Study 6.

We estimated confidence intervals for this difference with the/EMMEANS … COMPARE command in SPSS Mixed Models. The estimated mean difference of 59.92 has a confidence interval from 32.79 to 87.05 (Figure 2). After averaging the response latencies for each participant and condition, the effect size for congruency was estimated as η_p_^2^ = .338.

### Discussion

The goal of Study 4 was to validate ScriptingRT with a well know paradigm, the Stroop Effect. Participants had to decide which color a target series of letters was written in, responding to 60 trials. The results showed that responses on incongruent trials were about 60****ms slower than those on congruent trials, and that ScriptingRT measured this difference precisely enough to be significant in the sample of 19 participants.

## Study 5: Comparing ScriptingRT and DMDX in the Laboratory

Study 5 tested the precision of ScriptingRT by comparing it to DMDX [5] with both running on the same computer in a controlled laboratory setting.

### Method

#### Overview and design

All participants completed a Stroop task twice on the same PC, once in ScriptingRT and once in DMDX. The order of software was counterbalanced. The complete design was thus 3 (targets words, within) ×2 (color, within) ×2 (software: ScriptingRT vs. DMDX, within) ×2 (first task: DMDX vs. ScriptingRT, between).

#### Participants

Nineteen undergraduates students from a Portuguese university took part in the experiment and were compensated with a 5 € gift voucher (Age: *M* = 25.3, *SD* = 9.1). The data from one participant had to be excluded because 27 responses were either incorrect or outside of the response window. Thus, data from 18 participants were analyzed.

#### Materials and procedure

Participants were informed that we were testing several software packages and therefore they had to perform the same task twice. Between the two versions of the task there was a short break. Participants were run individually. The Stroop task was programmed as before and conducted in Portuguese. The DMDX procedure matched the one in ScriptingRT as closely as possible. In ScriptingRT, the size of the Flash app in the browser was set to 1366×768 pixels, with a frame rate of 24 Hz, run in Mozilla Firefox with the Adobe Flash plugin. In DMDX, the task was displayed with the same resolution, but DMDX used the standard frame rate of 60 Hz. This study was run in the laboratory on Sony Vaio Core i5 laptops, running Windows 7 (64-bit). These laptops have 13 inch screens, with a refresh rate of 60 Hz.

### Results

We first removed incorrect trials and trials with latencies above 1600****ms and below 300****ms (a total of 4.03%).

We submitted the individual response latencies to a mixed model, with congruency, software, order of software, and all their interactions as fixed effects, and participant as the grouping variable.

Congruent trials were faster than incongruent trials in both ScriptingRT (*M* = 629.66, *SD* = 234.17 and *M* = 729.04, *SD* = 301.44, respectively) and DMDX (*M* = 504.85, *SD* = 172.98 and *M* = 565.73, *SD* = 229.88, respectively). The main effect of congruency was significant in the mixed model, *F*(1,1358) = 21.23, *p*<.001. In addition, there was a main effect of software, *F*(1,1358) = 58.47, *p*<.001. DMDX recorded responses as faster, *M* = 551.98, *SD* = 201.38, than ScriptingRT did, *M* = 631.63, *SD* = 243.42. Importantly, the congruency effect was not moderated by software, *F*<1.

In addition there were some effects that are irrelevant given the present purposes. Software and order interacted, indicating that the second software run produced faster answers (presumably because of practice). We also found a moderation of the overall Stroop effect by which software was run first, which is most likely a randomization artifact.

Turning to confidence intervals of the estimated mean differences, we found that the difference due to congruency in ScriptingRT was 61.35, ranging from 33.96 to 88.75 (Figure 2). Responses in DMDX resulted in a smaller difference of 48.96, ranging from 21.59 to 76.33.

Effect sizes were computed after averaging latencies, for each participant, software, and congruency separately. We used two separate GLMs with congruency as a repeated measure. The effect size for congruency was η_p_^2^ = .37 in ScriptingRT and η_p_^2^ = .24 in DMDX.

### Discussion

Study 5 compared ScriptingRT and DMDX, using again the classic Stroop effect. All participants performed the task in both programs in the laboratory on the same computer. We found that the size of the Stroop effect was not affected by which software was used. If there was a difference at all, ScriptingRT showed larger interference effects than did DMDX. However, that ScriptingRT indicated significantly longer response latencies. The difference is close to the differences observed in Studies 1 and 2. Note that it seems impossible to state exactly how much measuring in Flash with ScriptingRT adds as a constant to the latency, as this seems to differ between browsers (Study 1) and presumably also depending on hardware [18].

## Study 6: Comparing ScriptingRT to Inquisit Web Edition, Running Online

### Method

#### Overview and design

Study 6 went beyond the previous studies by comparing ScriptingRT to the only currently available commercial solution for online data collection, namely Inquisit Web Edition. We ran this study online, directing voluntary participants to a website and assigning them randomly to either a ScriptingRT or an Inquisit Web Edition version of the same Stroop task that was used before. Software thus varied between participants in this study.

### Participants

Undergraduates of a Portuguese university were contacted by e-mail and asked to perform the experiment voluntarily. The E-mail contained a brief explanation of the experiment's goal (not mentioning interference) and a link to the web page. Each participant was assigned randomly to either ScriptingRT or Inquisit. Data from 43 participants were collected, but one had technical problems, leaving 42 in the final sample; 18 performed the task in Inquisit and 24 in ScriptingRT (Age: *M* = 25.8 *SD* = 7.78).

#### Materials and procedure

ScriptingRT used a resolution of 600×800 pixels. The frame rate in Flex was set to 60 Hz. The task was programmed in Inquisit and deployed using the web edition version. The experiment ran on the participant's computer with their specific resolution and frame rate.

### Results

Again, we first removed trials with incorrect responses and responses outside the 300 to 1600****ms time window (in total 6.9%). Individual latencies were subjected to a mixed model with software, congruency, and their interaction as fixed factors, and participant as grouping variable.

Congruent trials were faster than incongruent trials in both ScriptingRT (*M* = 569.16, *SD* = 224.79 and *M* = 630.10, *SD* = 269.74) and Inquisit (*M* = 526.58, *SD* = 184.26 and *M* = 576.24, *SD* = 250.66). The main effect of congruency was significant, *F*(1,1520) = 45.40, *p*<.001. Software had neither a main effect, nor did it interact with the congruency effect, both *F*s<1.

When we estimated means and confidence intervals, the congruency effect in ScriptingRT was estimated as 69.57, with an interval from 46.19 to 92.94. In Inquisit, the congruency effect was estimated as 51.82, with an interval from 25.32 to 78.32 (Figure 2).

We again averaged latencies for participants to two scores for congruent and incongruent trials, and then ran two GLMs to estimate effect sizes. The effect size in ScriptingRT was η_p_^2^ = .33, and in Inquisit η_p_^2^ = .28.

### Discussion

In an online study, we compared ScriptingRT to the only commercially available software that measures response latencies online. Again, the Stroop effect replicated in ScriptingRT, and again we found no difference from another software, this time Inquisit Web Edition. The latencies measured by ScriptingRT were again somewhat longer, but the difference was not significant here. Note that this study used a between subjects design, which added more error variance to the between software comparison.

## General Discussion

ScriptingRT is an open source software framework for developing online response latency studies running in Adobe Flash. ScriptingRT studies are programmed in Apache Flex, with a combination of four elements: (1) ScriptingRT-provided components (in MXML) that create the building blocks of a latency study (e.g., blocks, items, and frames), (2) standard Flex components (in MXML) that describe general content such as text, graphics, images, or sound, (3) additional Flex components that add custom components, and (4) programs (in ActionScript) that add custom functionality.

Flash has been used to conduct response latency studies before, notably using the IAT [7]. In the current paper, we show that the precision and accuracy provided by Flash in the form of ScriptingRT is not perfect, but suitable for many paradigms. As Figure 2 shows, using ScriptingRT, we replicated in three studies the classic Stroop interference effect with samples of around 20 participants. In two of those studies, we compared the combined interference and facilitation effect obtained in ScriptingRT to the same effect in a different software (DMDX, Inquisit Web Edition), without finding a significant difference. Figure 2 also shows that all five computed Stroop differences fall within the 95% confidence intervals of all other studies and conditions, suggesting a solid replication of the effect across software. Notably, when computing effect sizes, we found somewhat larger effects in ScriptingRT in all three comparison studies. Even though the differences between software were never significant, this assures that we did not simply have too little power in the tests of an inferior software, when measuring ScriptingRT against the competitors.

The solid replication and the missing significant differences across software might be surprising given the offsets documented in Studies 1–3. To understand this, one should keep in mind that the delays have a random distribution with a standard deviation that is much smaller than the studied effect itself. In addition, other sources of error variance, in particular due to participants, are distributed randomly. In the current paper, we did not address the question of how smaller experimental effects may hold up in the same comparison. In other words, can we expect to replicate response latency differences around 20 or even 10****ms? It can be expected that the smaller the effect, the more problematic the noise introduced by ScriptingRT (and online experimentation more generally). Both pilot testing and simulation can be used to estimate the impact on a particular paradigm with given number of trials and variance in materials. For researchers interested in using ScriptingRT to study smaller effects, we recommend to a) include conditions that replicate well known effects as a comparison condition, and b) use pilot studies to estimate effect sizes and required sample size for sufficient power.

Tests using special hardware designed to register the precise timing of stimulus presentations found evidence that as a stimulus presentation vehicle, Flash is not precise to the millisecond; we found constant lags of about 24****ms using the Flash-based ScriptingRT software. We also saw that compared to standard software packages, the standard deviation of the measures was larger; but because these deviations were below 5****ms, it still seems to be useful for most purposes. This amount of additional variance is comparable to what was standard for many years when regular keyboards were used. Finally, ScriptingRT overestimates the response latencies by a constant amount of about 60****ms. These issues must be taken into account when planning a study using ScriptingRT and interpreting results.

Importantly, variance added by measuring response latencies in Flash rather than a native software is only one source of added variance when collecting data online. In addition, it is quite likely that different implementations (e.g., Flash plugin vs. Flash support integrated into Chrome), different operating systems (e.g., Windows vs. OS X), additional programs running on the computer, and the quality of the hardware, can influence both the presentation timing and the time measurement, and will add error variance that is indistinguishable from variance due to interindividual differences. The present data do not yet allow conclusions on this beyond the differences found in Study 1, but as a precaution, any online data collection project should collect as much technical data on the client machine as possible. We will continue with formal tests to find out more about these variances.

With the current evaluation we show that ScriptingRT can replicate strong effects. What remains a task for future work is to provide exact guidelines for a priori judgments of how added noise in latency measures, combined with variability by assessing more diverse samples, will affect test power and the occurrence of false positive findings.

ScriptingRT can be used in its current form to implement many different paradigms, and it is under further development. Its source code is available, and we invite contributions to it. We will actively continue to develop it, document programmed enhancements online, and empirically test them.

## Conclusion

ScriptingRT is a software library that allows programming response latency studies using Apache Flex and Adobe Flash. ScriptingRT offers the building blocks for typical response latency paradigms in XML, while additional functionality can be added with programming a scripting language. The testing reported herein suggests that it is, in its present form, a viable software for using standard response latency paradigms online. Our evidence suggests that for a robust paradigm like Stroop, ScriptingRT is comparable to the other available options (i.e., Java, and HTML/JavaScript, and Inquisit Web Edition).

Future research will ideally run studies with a variety of software in a variety of environments, for instance using Flash and JavaScript to access large samples, native software running on a desktop computer and Java to get more precise measures, and accompanying replications in the lab with precise equipment. We see Flash and ScriptingRT as an important component in this mix, because it is free and accessible, and thus allows smaller labs to run large studies.

We contend that running response latency studies online can contribute to overcoming a number of problems that trouble current psychological research: First, many studies have low power. Here, easy access to participants online can help [15], [30], even though power will be slightly decreased by the additional noise from using Flash compared to native PC software. Second, many studies are conducted by sampling only from Western, educated, industrialized, rich, and democratic societies [31]. Running online naturally restricts sampling to educated and industrialized populations that have access to computers and the Internet, but it may go a long way towards getting more cultural variability into cognitive and social science studies. Finally, replication is becoming increasingly important in psychological research [32], [33]. Response latency studies may be especially difficult to replicate because their material is often programmed in proprietary software, and not easily shareable. ScriptingRT is completely open source, and its source files are simple text, and thus easily shareable.

ScriptingRT is already being used by various researchers to run studies. We believe it is quite accessible for the average researcher. Because ScriptingRT is an open source software program, developed to be used in conjunction with other open source programs, we look forward to its growth in many directions, with wide applications. The results obtained and reported in this article and the expansion possibilities allowed by the software's open source nature, make us confident that ScriptingRT will gain researchers' interest and we hope that its usage will be widespread.
